# Highlight on the dynamic organization of the nucleus

**DOI:** 10.1080/19491034.2016.1243634

**Published:** 2016-10-07

**Authors:** Stephen D. Thorpe, Myriam Charpentier

**Affiliations:** a Institute of Bioengineering, School of Engineering and Materials Science, Queen Mary University of London, London, UK; b Cell and Developmental Biology, John Innes Center, Norwich, UK

**Keywords:** chromatin organization, gene expression, LINC complex, lamin, nucleus, nuclear architecture, nucleopore, nuclear movement

## Abstract

The last decade has seen rapid advances in our understanding of the proteins of the nuclear envelope, which have multiple roles including positioning the nucleus, maintaining its structural organization, and in events ranging from mitosis and meiosis to chromatin positioning and gene expression. Diverse new and stimulating results relating to nuclear organization and genome function from across kingdoms were presented in a session stream entitled “Dynamic Organization of the Nucleus” at this year's Society of Experimental Biology (SEB) meeting in Brighton, UK (July 2016). This was the first session stream run by the Nuclear Dynamics Special Interest Group, which was organized by David Evans, Katja Graumann (both Oxford Brookes University, UK) and Iris Meier (Ohio State University, USA). The session featured presentations on areas relating to nuclear organization across kingdoms including the nuclear envelope, chromatin organization, and genome function.

## Dynamic organization of the plant nucleus

### Nuclear envelope and nucleoskeleton

**Figure 1.**
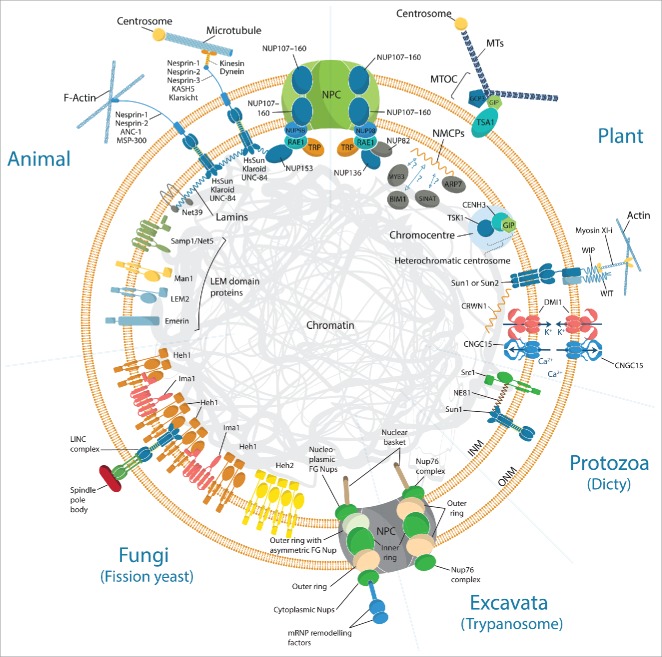
Highlight on functional components of the nuclear envelope across kingdoms. The fundamental units of the LINC complexes which mechanically couple the nucleoskeleton and cytoskeleton are KASH (Klarsicht, ANC-1 and SYNE homology) domain-containing nesprins and SUN (Sad-1 and UNC-84) domain-containing proteins. Although functionally conserved, most of those components identified in metazoans do not have sequence homologues across kingdoms. With the exception of SUN proteins (e.g., SUN1, SUN2 and SUN3), KASH proteins (e.g. WIP, WIT) present no homology with their animal functional equivalent (e.g., Nesprin, ANC-1, Klarsicht, UNC-83, KASH5 or MSP-300). Similarly, most of the inner nuclear envelope proteins gathered in the LEM2-emerin-MAN1 (LEM) domain protein family, do not yet have functional equivalent in plant. The LEM domain proteins share an ability to bind lamins and tether repressive chromatin at the nuclear periphery. These have homologues in *Dictyostelium discoideum* (Src1) and yeast (Heh1, Heh2 and Ima1) which in the absence of lamins, play a role in nuclear stiffening through chromatin tethering to the INM. The functional lamin-like proteins in plant are the nuclear matrix constituent proteins (NMCPs) also called crowded nuclei (CRWN) in *Arabidopsis*, while NE81 has been identified in protozoa. SUN1 and SUN2 bind CRWN1, whereas NMCP1 has several putative interactors including ARP7. In *M.* truncatula, the ion channel complex DMI1-CNGC15 which localize to both inner nuclear membrane (INM) and outer nuclear membrane (ONM), are required for symbiotic factor induced nuclear localized calcium release. At the nuclear pore complex (NPC), plant-specific FG-repeat nucleoporin, NUP136 and NUP82, have been identified. While the trypanosome NPC is predominantly symmetric, the yeast NPC is comparatively less so.

While often structurally and functionally similar, the composition of the nuclear envelope across kingdoms is surprisingly diverse (Fig. 1). For example, the nucleoskeleton meshwork that underlies the inner nuclear membrane and structurally supports the nuclear envelope, the lamina, is formed of lamin proteins in metazoans and potentially of the nuclear matrix constituent proteins (NMCPs) in plants.^1^ Despite the lack of sequence similarity with lamin proteins, the NMCPs are structurally similar and equivalently required for the regulation of nuclear shape, size and heterochromatin organization. The metazoan lamina is anchored to the nuclear envelope, nuclear pore complexes (NPCs) and chromatin via lamin-binding proteins, some of which regulate signaling and transcription. No orthologues of the metazoan lamin interactors can be found in plants with the exception of Sad1 and UNC84 (SUN) domain proteins.^2^ The characterization of the NMCP binding proteins represents an important focus to understand plant nuclear dynamics. Using far-western blotting and yeast 2 hybrid approaches, Daisuka Tsugama (Hokkaido University, Japan) identified new putative interactors of *Daucus carota* NMCP1. These interactors include the nuclear localized actin related protein 7 (ARP7)^3^ and 3 putative nuclear proteins, MYB-type transcription factor 3 (MYB3), C3HC4 RING-finger proteins (SINAT) and a microtubule binding protein (BIM1). These proteins are strong candidates for *in vivo* DcNMP1-binding proteins to chromatin, and might be involved in chromatin regulation and mechanotransduction.

The NPCs establish an essential conduit for nucleo-cytoplasmic transport, which contribute to basic cellular activity and cellular responses to biotic and abiotic stresses.^4^ NPCs are composed of approximately 30 different proteins called nucleoporins (NUPs), which can be subdivided into 4 classes: transmembrane, core scaffold, linker and Phe-Gly (FG)-repeat NUPs.^5,6^ In metazoan, NPCs are anchored to the lamina via direct interaction with a FG-repeat NUP, NUP153. The plant functional homolog of NUP153 is believed to be NUP136. Similarly to NUP153, NUP136 is a mobile nucleoporin which interacts with the RNA export factor 1 (RAE1) and is required for nuclear morphology.^7^ Additionally, Kentaro Tamura (Kyoto University, Japan) discovered a new plant-specific NUP136 homolog, called NUP82 based on its molecular mass. Immunoprecipitation experiments reveal that NUP82 interacts with NUP136 and RAE1, potentially contributing to gene expression and NPC anchoring via direct binding to NMCPs. The *Arabidopsis thaliana* double mutant *nup82nup136* is impaired in defense and salicylic acid (SA) induced gene expression, resulting in defects in SA signaling and immune responses to *Pseudomonas syringae* pv. Tomato DC3000. Previously, constituents of the core scaffold of the NPCs, the NUP107–160 sub-complex, were shown to be required for innate immunity and responses to root legume symbiotic microorganisms.^8,9^ The research performed by Kentaro Tamura reinforced the role of plant NPCs in plant microbe interactions, not only via the NUP170–160 sub-complex but also via the FG-repeat NUPs.

### Chromocenter dynamics

In plants, NMCP is connected with cytoskeletal elements through the linker of nucleoskeleton and cytoskeleton (LINC) complex.^2^ The LINC complex is composed of SUN and Klarsicht/ANC-1/Syne-1 homology (KASH) proteins which span the double membrane of the nuclear envelope and mechanically couple the nucleoskeleton and cytoskeleton (Fig. 1). At the inner nuclear membrane, the LINC complex is anchored to NMPC via SUN proteins.^2^ While plant LINC complexes have been shown to regulate various nuclear processes including nuclear morphology, migration and meiotic chromosome organization,^10^ their role in chromatin regulation still remains poorly understood. In *A. thaliana* interphase nuclei, clusters of heterochromatin, often referred to as chromocenters, can be found at the nuclear periphery, where they are typically associated with transcriptionally repressive chromatin.^11^ Using the software NucleusJ,^12^ Axel Poulet (Oxford Brookes University, UK & Clermont University, France) reported that chromocenters are positioned in close proximity to the periphery of the nucleus but that this distance varies with the nuclear volume in *A. thaliana* LINC complex and NMCP mutants. Additionally, using 3D-FISH and analyses of the centromeric and peri-centromeric short repetitive DNA, he demonstrated that both LINC complex and NMCP are required for heterochromatin organization, compaction and associated with the release of gene silencing. Altogether, this suggests that the LINC complex and NMCP contribute to the regulation of heterochromatin organization and gene expression potentially via the indirect interaction of unknown components. Several improved nuclear proteomics approaches presented by Kentaro Tamura (Kyoto University, Japan) using *A. thaliana*, and Beata Petrovska (Institute of Experimental Botany AS CR, Czech Republic) using Barley,^13^ may help identify novel candidates to decipher the complex molecular regulation of chromatin at the nuclear periphery.

In metazoan, at the core of the chromocenter during interphase, the centromere is compact with a specific histone variant CenH3 whose deposition is tightly controlled by specific histone chaperones; no chaperone has yet been identified in plants. In related studies, microtubule regulators associated with the centromere have emerged as strong candidates.^14,15^ The small γ-tubulin complex component 3 (GCP3)-interacting proteins (GIPs) co-immunoprecipitate with the centromere-histone H3 variant CENH3, and co-localize with the chromocenters and CENH3 at the nuclear periphery in interphase nuclei.^15^ In *A. thaliana*, the *gip1gip2* double mutant exhibits a decreased level of centromeric proteins including CENH3, strong microtubule disruption as well as deformed nuclei and abnormal distribution of NPCs and SUN1.^15,16^ These results indicate that GIPs are required not only for the recruitment and/or stabilization of CENH3 and centromeric cohesion but also for nuclear morphology and nuclear envelope organization. Marie-Edith Chabouté (IBMP, CNRS Strasbourg, France) previously revealed that GIPs interact with the TonSoKu (TSK)-associating protein 1 (TSK1) interactor (TSA1).^14,17^ The Tsk1 mutant is sensitive to DNA-damage agents and presents reduced epigenetic gene silencing suggesting that TSK1 is required for genome maintenance.^18,19^ Marie-Edith Chabouté et al. explored the genetic relation between GIP1, GIP2 and TSK1. Using neutral comet assays and a cytological marker of double strand breaks, she revealed that the double mutants *gip1tsk2* and *gip1gip2* both present an increase of double strand breaks close to heterochromatin. Additionally, the results of FISH and immuno-cytological approaches proved that similarly to that for GIPs, TSK2 is required for centromeric cohesion. Further investigation will be required to clarify the epigenetic relation between GIPs and TSK, specifically whether some of them may interact in complexes that affect the transcriptional status at the chromocenters.

### Chromatin structure-function

Packaging of DNA into chromatin allows eukaryotes to contain large volumes of DNA into a small nuclear volume, to protect the DNA structure and sequence as well as control gene expression and DNA replication. These functions are regulated by changes in nucleosome positions and chromatin structures via complex mechanisms that are not yet fully understood. One of the regulatory mechanisms of gene transcription at the chromatin level is the control of accessibility of transcription machinery to appropriate DNA sequence. Hank Bass (Florida State University, USA) reported on chromatin structure-function relationships in the maize nucleus at multiple spatial scales. A differential nuclease sensitivity sequencing assay was developed based on the micrococcal nuclease (MNase) digestion to discover open chromatin regions in the maize genome. The MNase hypersensitive sites discovered were functionally verified by genome-wide association studies to explain most of the heritable phenotypic variation in maize.^20,21^ At a larger scale, in maize root tip cells, 3D quantitative analysis of spatio-temporal DNA-replication patterns unexpectedly revealed 2 intermingled chromatin compartments distinguished by their replication timing (early versus middle S phase) and degree of 4′6-diamidino-2-phenylindole-stained condensation.^22^ A mini-domain replication model from this work needs to be tested via future experiments.

### Nucleus positioning and movement

The nucleus is anchored to the cytoskeleton by specific interactions at the outer nuclear membrane via the LINC complex. These interactions anchor and regulate the movement of the nucleus in cells, a process known to be tightly coupled to certain plant physiological processes.^23^ In guard cells, the KASH protein, spectrin repeat containing nuclear envelope protein 1 (SINE1), binds actin filaments directly to position the nucleus in the cell center.^24^ In mesophyll cells, nuclear shape and dark-induced movement is regulated by the KASH proteins, tryptophan-proline-proline (WPP) domain-interacting proteins (WIP), and the WPP domain–interacting tail-anchored protein (WIT) via recruitment of myosin XI-i.^25^ Iris Meier (Ohio State University, USA) presented the recent discovery that the LINC complex is also required for successful reproduction in *A. thaliana*. She demonstrated that the SUN and KASH protein (WIP-WIT) complexes are required for the movement of the pollen vegetative nucleus during pollen tube growth as well as for pollen tube ovular guidance and reception.^26^ Altogether, the function of LINC complexes in plant nuclear anchorage is unambiguous, although other nuclear components such as the NPCs might play a role. For instance, Kentaro Tamuro (Kyoto University, Japan) identified the gene *KAKU3* in forward genetic screen for mutants impaired in nuclear movement. The *kaku3* mutant fails to anchor the nucleus during cytoplasmic streaming observed in live hypocotyl cells. The *KAKU3* gene is thought to encode a nuclear protein linked to the NPC, yet mutations in other NPCs did not show anchorage defects, which suggests a specific function of KAKU3. Further studies might shed light on how the NPC regulates nuclear anchorage as well as the relation to nucleocytoplasmic transport and gene regulation. While less studied, several other processes are tightly coordinated with nuclear movement e.g. root hair tip growth and trichome development as well as symbiotic and pathogenic plant-microbe interactions.^23^ To understand the role of nuclear movement in those processes, the identification of the nuclear proteins involved is required. Myriam Charpentier (John Innes Center, UK) presented the recent discovery of new nuclear envelope localized calcium channels encoded by 3 cyclic nucleotide gated channel 15 (CNGC15) in *Medicago truncatula*.^27^ CNGC15s are located at the inner and outer nuclear membranes where they interact with a potassium permeable channel (DMI1) to orchestrate the generation of symbiotic factor-induced nuclear-localized calcium oscillations (Fig 1).^27^ Nuclear localized calcium oscillations are one of the fastest physiological responses occurring at plant root cell nuclei upon perception of diffusible symbiotic factors.^9^ These calcium oscillations are essential to activate the endosymbiotic program. Interestingly the perception of the symbiont by the host cells induces, concomitantly to the nuclear calcium oscillation, the repositioning of the nuclei toward the site of penetration.^28^ Whether the calcium oscillation and the nuclear repositioning are connected is unclear and will require further investigation.

### Dynamic organization of non-plant nuclei

#### Lamin A/C regulated nuclear mechanics and genome function

One key theme which emerged from this session was an appreciation of the interplay between nuclear organization and the cell's biophysical environment. Diseases involving mutations in nuclear envelope protein components often manifest themselves in tissues subjected to mechanical stress.^29^ Yosef Gruenbaum et al. (Hebrew University of Jerusalem, Israel) presented a study investigating the effects of various Emery-Dreifuss muscular dystrophy (EDMD) associated mutations on the response of nuclei in living *Caenorhabditis elegans* exposed to mechanical strain. The EDMD lamin mutation L535P increased the resistance to strain specifically in muscle nuclei. This mechanical response could be rescued through inhibition of lamin prenylation via depletion of farnesyl diphosphate synthase gene (*fpds-1*), which also reversed the muscle phenotypes restoring normal mobility; and provides a potential future therapeutic approach for EDMD.^30^ Interestingly, this also induced a small shift in lamin distribution from the nuclear periphery to the nucleoplasm. Roland Foisner et al. (Medical University Vienna, Austria) demonstrated a novel role for nucleoplasmic lamin A/C in regulating euchromatin. This nucleoplasmic lamin A/C directly binds euchromatin and is associated with chromatin binding protein lamina-associated polypeptide (LAP)2α. Lamin A/C in LAP2α deficient cells is absent from euchromatic regions and its absence was associated with a change in epigenetic histone marks in euchromatin.^31^ Furthermore, overexpression of LAP2α in cells expressing progerin, a lamin A mutant causing the premature aging disease Hutchinson Gilford Progeria Syndrome (HGPS), could rescue proliferation and the expression of extracellular matrix (ECM) genes.^32^ Reorganisation of Lamin A/C toward the nuclear periphery was also observed in stem cell differentiation by Stephen Thorpe et al. (Queen Mary University of London, UK). While lamin A/C phosphorylation increased with differentiation, strain application reduced phosphorylation. Defects in lamin A/C processing are associated with disease and aging. Prelamin A accumulation has been observed in dilated cardiomyopathy (DCM), and Daniel Brayson et al. (King's College London, UK) observed via a transgenic mouse with cardiomyocyte specific prelamin A accumulation, a rapid induction of DCM with mice succumbing by 6 weeks. This was associated with disruption of the LINC complex, DNA damage and senescence. Senescence and prelamin A accumulation have also been associated with the occurrence of nucleoplasmic reticulum.^33^ However, the mechanism behind these invaginations in the nuclear envelope is unclear. David Vaux et al. (University of Oxford, UK) have found that both membrane phospholipid and associated lamin components of these nuclear envelope invaginations are newly synthesized, suggesting an active process of invagination rather than external deformation of pre-existing nuclear envelope. The role of nucleoplasmic reticulum is unclear; while it may act as a deep nuclear calcium signaling source,^34^ it may also provide a reservoir of nuclear membrane to facilitate conservation of volume while the nucleus distorts.

### Nuclear migration and the LINC complex

The LINC complex, which bridges the nuclear envelope, plays a role in nuclear movement through its association with different cytoskeletal components. Stephen Thorpe et al. (Queen Mary University of London, UK) demonstrated that nuclear orientation is offset from that of the actin cytoskeleton in differentiating stem cells subjected to uniaxial strain, and this is associated with an increase in both SUN1 and SUN2 expression. Daniel Starr (University of California, Davis, USA) presented an elegant study of nuclear migration in *C. elegans* P cells, where the nucleus (3–4 µm diameter) must migrate through a 150 nm space between the body wall muscle and the worm's cuticle. Interactions between canonical SUN and KASH proteins, UNC-84 and UNC-83 (microtubule recruitment) or ANC-1 (actin recruitment), are key to the switch between nuclear anchorage or migration (Fig. 1).^35^ In the P cell model, nuclei move toward the minus ends of microtubules using dynein. This is the opposite to what was previously observed in embryonic hyp7 cells.^36^ However, both kinesin and actin cables assist in squeezing the P cell nucleus though this constriction while the nuclear lamina completely rearranges to facilitate extreme nuclear distortion.

### Nuclear envelope proteins and chromatin tethering

In addition to the *C. elegans* model described above (Yosef Gruenbaum et al.), nucleus distortion in response to an external mechanical perturbation was also investigated in both mesenchymal stem cells and in yeast. In both cases, nuclear deformation was regulated, at least in part, by chromatin state. Stephen Thorpe et al. (Queen Mary University of London, UK) demonstrated increased chromatin compaction in differentiated stem cell nuclei, which was associated with a reduction in nuclear elongation in response to strain. However, extensive lamin A/C reorganisation was also observed. Megan King (Yale School of Medicine, USA) presented a study in fission yeast, which lack a nuclear lamina, to investigate how chromatin tethering to the nuclear envelope influences nuclear stiffness. Optical tweezers were used to perturb isolated yeast nuclei with and without inner nuclear membrane chromatin tethers Heh1, Heh2 (both orthologues of mammalian LEM domain proteins Man1, LEMD2 and emerin; Fig. 1) and Ima1 (homologous to mammalian Net5/Samp1). Nuclei lacking tethers were less stiff and exhibited increased chromatin flow associated with a reduction in viscosity, particularly in frequency ranges which recapitulate the kinetics of cytoskeletal dynamics.^37^ Additionally, *in vivo* fluctuations in nuclear morphology were observed to be driven by microtubules. King et al. also observed co-localization of Heh1 and Ima1 with *S. pombe* SUN domain protein Sad1 which resides at the centromere-spindle pole body interface of the nuclear envelope. This accumulation of chromatin tethers was associated with a large area of heterochromatin. In another unicellular organism, *Dictyostelium discoideum*, Ralph Gräf et al. (Universität Potsdam, Germany) observed an enrichment of SUN1 in the region of the spindle-pole body interface of the nuclear envelope. SUN1 was observed to interact with a lamin-like protein, NE81,^38^ which also associated with and required Src1 (homologous to MAN1) for localization at the nuclear envelope. The tissue specific and postnatal role of MAN1 in angiogenesis, heart development and muscle regeneration was explored by Alexander Stubenvoll et al. (Max-Planck Institute for Heart and Lung Research, Germany) using a conditional knock out mouse model. Loss of MAN1 resulted in severe cardiac abnormalities and was embryonic lethal. MAN1 also plays a key role in muscle homeostasis and regeneration which was disrupted with loss of MAN1 in muscle stem cells, although inhibition of TGF-β signaling rescued these defects. The association of LEM domain proteins with key signaling pathways was also highlighted by Parisa Ghanbari et al. (Max-Planck Institute for Heart and Lung Research, Germany) who identified a role for emerin in the restriction of Wnt/β-catenin signaling,^39^ suggesting that the absence or abnormal localization of emerin leads to hyper-activation of this pathway and genomic instability contributing to cancer initiation.

### Evolution and the nuclear envelope

The nuclear envelope is the defining structure of the eukaryotic cell, and its most prominent structures include the nuclear pore complexes (NPCs) and the closely associated filamentous nuclear lamina (Fig. 1). Mark Field (University of Dundee, UK) presented an overview of the evolution of the NPC based on interactome mapping of the trypanosome NPC, a representative, highly divergent eukaryote. While retaining similar protein composition, considerable architectural dissimilarities exist between opisthokont (yeast and metazoans) and excavate (trypanosome) NPCs.^40^ Interestingly, although lamins were assumed a derived feature of the animal nucleus, they found lamin homologs with shared domain architecture and sequence motifs in diverse protists.^41^ This ancient evolutionary origin of nuclear lamins was further substantiated by Ralph Gräf et al. (Universität Potsdam, Germany) who observed the presence of a lamin-like protein, NE81, in another unicellular organism, *Dictyostelium discoideum*.^38^

### Chromatin organization and genome function

In addition to involvement in signaling pathways, nuclear envelope proteins direct establishment of genome-wide patterns of peripheral heterochromatin formation. Eric Schirmer (University of Edinburgh, UK) presented work demonstrating that tissue-specific nuclear envelope gene tethering does indeed provide an additional layer of genome regulation. Muscle-specific nuclear transmembrane proteins (NETs; Fig. 1), NET39, Tmem38A and WFS1 direct specific myogenic genes to the nuclear periphery to facilitate their repression.^42^ While genes were repressed in the absence of NET gene tethering, this repositioning contributes between ^1^/_3_ and ^2^/_3_ of a gene's normal repression in myogenesis. Furthermore, expression of tissue-specific NETs from muscle, liver and fat in a fibroblast cell line was able to recapitulate gene repression specific to the tissue of NET origin. Sequencing of 74 unlinked EDMD patients revealed several of these spatial genome organization NETs from muscle as strong disease candidates. The mechanism through which chromosome and gene movement occurs remains elusive. Joanna Bridger et al. (Brunel University, UK), in a snail cell model of host-parasite interactions, revealed that specific chromosomes and genes move rapidly (15 min.) to a new non-random location.^43^ This movement is associated with expression as the actin gene locus was observed to move ∼30 min. before transcription was detected. This active and directed movement is dependent on nuclear actin and myosin polymerisation. In addition to silencing and activation, the positioning of gene loci and chromosomes also facilitates interactions between different gene regions. Limb specific sonic hedgehog gene (*Shh*) expression is regulated by the enhancer designated ZRS, ∼1 Mb away. Iain Williamson et al. (MRC Institute of Genetics and Molecular Medicine, UK), using super-resolution microscopy with fluorescent in situ hybridization (FISH) and chromatin conformation capture (5C),^44^ identified elevated frequencies of *Shh*/ZRS co-localization consistent with the formation of an enhancer-promoter chromatin loop in *Shh* expressing regions of limb bud only.^45^ However close *Shh*/ZRS proximity in the nucleus occurs regardless of whether the gene or enhancer is active. This constrained chromatin configuration may enhance the opportunity for the active enhancer to locate and instigate *Shh* expression.

### Conclusion and future perspectives

Overall the research presented at this meeting demonstrated how fast this field has moved forward in recent years. Research across kingdoms on the dynamic organization of the nucleus is undergoing a shift in focus from fundamental knowledge of nuclear composition and connections, to understanding of the mechanisms and function of these components; instances of which include signaling roles of nuclear envelope proteins, nuclear envelope structural changes in disease, and the role of nuclear structure in epigenetics. Recent discoveries of nuclear components in lesser studied species and kingdoms (e.g., trypanosomes and *Dictyostelium*) has highlighted how much remains to be determined relating to nuclear composition and function in all species. As such, the discovery of CNGC15 as a plant nuclear-localized calcium channel represents a major breakthrough, paving the way to understand the regulation and genome function of biotic stresses-induced nuclear calcium signaling. In addition to new components, new roles for some of the previously discovered nuclear components in the regulation of a host of cellular processes are being discovered. While the components often differ substantially, discoveries in one kingdom can inform those in another. The NPC is one area where comparison across kingdoms has already proved highly informative.^40^ Evident at this meeting was a focus on the role of mammalian LEM domain proteins Man1, LEMD2 and emerin and their orthologues in *S. pombe* and *Dictyostelium*. The future looks bright in this emergent field of nuclear dynamics. This meeting has served to highlight, not just the differences, but the great degree of similarity in nuclear organization across kingdoms. As we shift our focus toward mechanism and function, the value of parallels drawn across kingdoms will be come all the more apparent.
